# Crystal Structure of Cytochrome P450 (CYP105P2) from *Streptomyces peucetius* and Its Conformational Changes in Response to Substrate Binding

**DOI:** 10.3390/ijms17060813

**Published:** 2016-05-25

**Authors:** Chang Woo Lee, Joo-Ho Lee, Hemraj Rimal, Hyun Park, Jun Hyuck Lee, Tae-Jin Oh

**Affiliations:** 1 Division of Polar Life Sciences, Korea Polar Research Institute, Incheon 406-840, Korea; justay@kopri.re.kr (C.W.L.); hpark@kopri.re.kr (H.P.); 2 Department of Polar Sciences, University of Science and Technology, Incheon 406-840, Korea; 3 Department of BT-Convergent Pharmaceutical Engineering, Sunmoon University, Asansi 336-708, Korea; shadowjhl@empal.com (J.-H.L.); rimalhem@gmail.com (H.R.)

**Keywords:** cytochrome P450, crystal structure, *Streptomyces peucetius*, X-ray crystallography

## Abstract

Cytochrome P450 monooxygenases (CYP, EC 1.14.14.1) belong to a large family of enzymes that catalyze the hydroxylation of various substrates. Here, we present the crystal structure of CYP105P2 isolated from *Streptomyces peucetius* ATCC27952 at a 2.1 Å resolution. The structure shows the presence of a pseudo-ligand molecule in the active site, which was co-purified fortuitously and is presumed to be a biphenyl derivative. Comparison with previously determined substrate-bound CYP structures showed that binding of the ligand produces large and distinctive conformational changes in α2–α3, α7–α9, and the *C*-terminal loop regions. This structural flexibility confirms our previous observation that CYP105P2 can accommodate a broad range of ligands. The structure complexed with a pseudo-ligand provides the first molecular view of CYP105P2–ligand interactions, and it indicates the involvement of hydrophobic residues (Pro82, Ala181, Met187, Leu189, Leu193, and Ile236) in the interactions between hydrophobic ligands and CYP105P2. These results provide useful insights into the structural changes involved in the recognition of different ligands by CYP105P2.

## 1. Introduction

Cytochrome P450 monooxygenases (CYPs) constitute a superfamily of heme-containing enzymes that are widely distributed among all biological kingdoms; they catalyze the hydroxylation of a wide variety of substrate molecules [1,2]. They are involved in the metabolism of xenobiotic compounds such as antibiotics and environmental toxins, and play vital roles in endogenous biosynthetic pathways, including those of sterols, fatty acids, prostaglandins, vitamins, and hormones [3,4]. CYPs have very strict substrate specificities, and catalyze regio- and stereo-specific reactions such as N-, O-, and S-dealkylations, deamination, dehalogenation, desulfuration, epoxidation, N-oxidation, peroxidation, and sulfoxidation [5]. Despite differences among the functions of various CYPs, their structural folds, binding modes to redox partners, substrate recognition, activation of dioxygen, and catalytic mechanisms are almost identical [6,7].

Actinomycetes produce two-thirds of the microbially derived secondary metabolites used in veterinary medicine [8]. *Streptomyces* is the largest genus of the Actinobacteria. *Streptomyces* species produce approximately 65% of all antibiotics, and their products include antibacterial, antifungal, antiparasitic, and anticancer agents, and immunosuppressants [9,10]. Many of these products are synthesized by polyketide synthases, and they are structurally modified by numerous biosynthetic enzymes, including CYPs, encoded by gene clusters [11]. *Streptomyces* genomes have also provided a rich source of CYP enzymes. For example, *S. coelicolor* contains 18 CYPs [12], *S. avermitilis* contains 33 CYPs [13,14], and *Mycobacterium tuberculosis* contains 20 CYPs [15]. *Streptomyces* CYPs generally have practical advantages for biotechnical applications because of their modification behaviors and abilities to generate a variety of novel compounds [16,17]. The most prevalent CYP families found in many *Streptomyces* species are the CYP107 and the CYP105 families. In many cases, genes encoding CYP107s are located in an antibiotic biosynthesis gene cluster, and their putative role is oxidative tailoring of the antibiotic during the final biosynthetic step [18]. CYP105s are the only CYP family that have been conserved with representatives in every *Streptomyces* species thus far investigated [19]. CYP105s include at least 17 subfamilies, with broad catalytic activities across a diverse range of substrates. CYP105s carry out biotransformation or degradation of xenobiotics, and biosynthesis of specialized bioactive molecules, including several antibiotics; this accounts for the fact that there is considerable structural variation within this family [20].

The structures of a number of CYPs have been solved using X-ray crystallography, and structure–function studies have shown that the heme group and active site are buried deep within the protein. The results suggest that CYP changes conformation dynamically to permit the substrate to enter the heme pocket via a ligand access channel. The overall structures and amino acid sequences of CYPs are similar to each other. However, significant structural differences are observed in the substrate-binding site and around the active site. For example, CYP105P1 has a very long and hydrophobic substrate-binding pocket for interaction with filipin I [21], and CYP105D7 contains two diclofenac-binding sites with a double-ligand-binding mode [22]. The CYP105P1 and CYP105D6 structures also exhibit significant differences between ligand-bound and ligand-free states, and in the BC loop and FG helices [21].

There are 21 known CYPs in the genome of *S. peucetius* but no significant role of CYP105P2 has been reported in the gene cluster of doxorubicin. Therefore, CYP105P2 is generally considered to be an independent monooxygenase. Previously, our group showed that *Escherichia coli* cells containing CYP105P2 and redox partner genes (*camA* and *camB*) acquire flavone hydroxylase activity, producing hydroxylated flavones in 10.35% yield [23]. The crystal structure was determined (2.1 Å resolution) to extend our knowledge of the molecular mechanism of CYP105P2 catalysis. A ligand resembling the biphenyl compound bisphenol A was unexpectedly found in the substrate-binding cavity; this ligand was probably incorporated during protein expression in *E. coli*. Although one CYP involved in bisphenol A degradation, from *Sphingomonas bisphenolicum* strain AO1, has had its molecular properties characterized [24], until now there have been no reports concerning the structural features associated with the hydroxylation of bisphenol A or related biphenyl compounds. This paper reports the structure of CYP105P2 from *S. peucetius* in a ligand-bound state, and compares that structure with the previously determined structures of other CYPs in ligand-bound and ligand-free forms. The resulting structure differs significantly from the other CYP structures and provides an alternative model for understanding substrate binding to CYP105P2, as well as demonstrating the mobile active site architecture of this enzyme.

## 2. Results and Discussion

### 2.1. Enzymatic Role of CYP105P2 and Bioinformatics Analysis

We tested whether this CYP105P2 enzyme can participate in this oxidative reaction by performing *in silico* analysis on a gene from *S. peucetius* ATCC27952 encoding a putative CYP, to verify the likely function of the responsible gene. We used Accelrys Discovery Studio software (version 3.5, Accelrys, San Diego, CA, USA) to construct homology models of CYP105P2, and then assessed their stereochemical properties and side-chain environments. Finally, a model flavone complex was used to validate the active site architecture, and structurally important residues were identified by subsequent characterization of the secondary structure [25]. In addition, the gene encoding CYP105P2 was expressed in *E. coli*, which contained a two-vector system carrying genes encoding putidaredoxin reductase (*camA*) and putidaredoxin (*camB*) from *Pseudomonas putida* for efficient electron transport. A whole-cell bioconversion reaction using the recombinant bacterial strains gave a yield of 10.35%. Finally, we verified that CYP105P2 is a bacterial flavone hydroxylase, with potential for use as a biocatalyst [23]. Here, we report our study of the structure of CYP105P2, which is involved in flavone hydroxylation.

Many other CYP enzymes belonging to the CYP105 subfamily are found among the Actinomycetes, and these oxidize a wide array of diverse substrates. To date, the crystal structures of five CYP105 members with diverse catalytic activities have been solved, providing important insights into their structure–function relationships. Examples are: MoxA (CYP105AB3) from the xenobiotic degradation pathway in *Nonomuraea recticatena* [26]; CYP105A1 (P450SU-1), which is involved in 1α,25-dihydroxyvitamin D3, from *S. griseolus* [27]; CYP105D6 and CYP105P1 from the filipin biosynthetic pathway in *S. avermitilis* [28]; and CYP105N1, which is involved in the cryptic coelibactin biosynthesis pathway, from *S. coelicolor* [18]. The gene encoding CYP105P2 has a G + C content of 70.5%, and the protein contains 399 amino acids (GenBank accession number: CAE53708). Our sequence analysis showed that CYP105P2 shares a high identity (92%) with CYP105P1 from *S. avermitilis*, and that it contains a dioxygen-binding region and a heme-iron-binding region, both of which are typical motifs of CYPs [23].

### 2.2. CYP105P2 over-Expression, Purification, and Spectral Analysis

**Figure 1:**
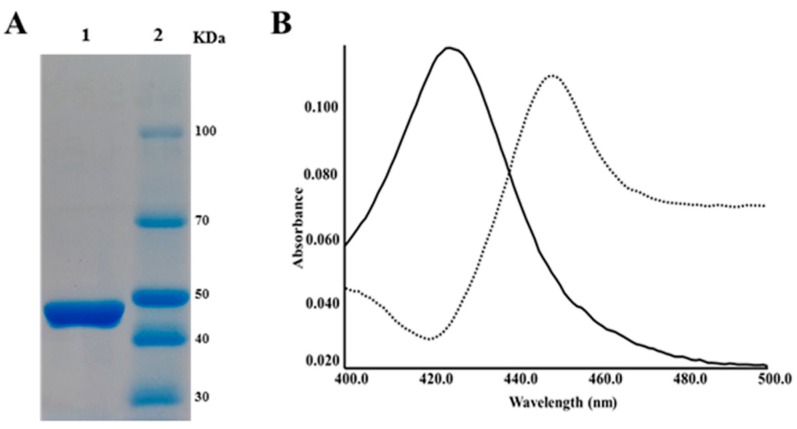
(**A**) Purification of CYP105P2: **Lane 1**, purified CYP105P2; **Lane 2**, molecular marker; (**B**) CO-reduced spectra of the heterologously overexpressed CYP105P2. Oxidized form, solid line; CO-reduced form, dotted line.

Based on the procedure for over-expression of *S. peucetius* CYPs previously reported by our group [23,29], high levels of correctly folded CYP105P2 were produced when over-expressed heterologously in *E. coli*. Purified CYP105P2 in the elution buffer was concentrated and desalted using a centrifugal filter (Amicon, Centricon-30). The final concentration of purified CYP105P2 was 6.63 mg/mL. SDS-PAGE analysis showed a single homogeneous band with the predicted molecular mass of 48 kDa (Figure 1A). Reduction of the hemoprotein from the ferric (Fe^3+^) to ferrous (Fe^2+^) form was achieved by addition of sodium dithionite to the sample (and reference cuvettes), and by passing CO through the sample cuvettes only. Reduction with CO produced a shift in the spectrum of CYP105P2 by about 30 nm, indicating that the electron density distribution on the heme group was significantly perturbed (Figure 1B).

### 2.3. Structure of CYP105P2

**Figure 2:**
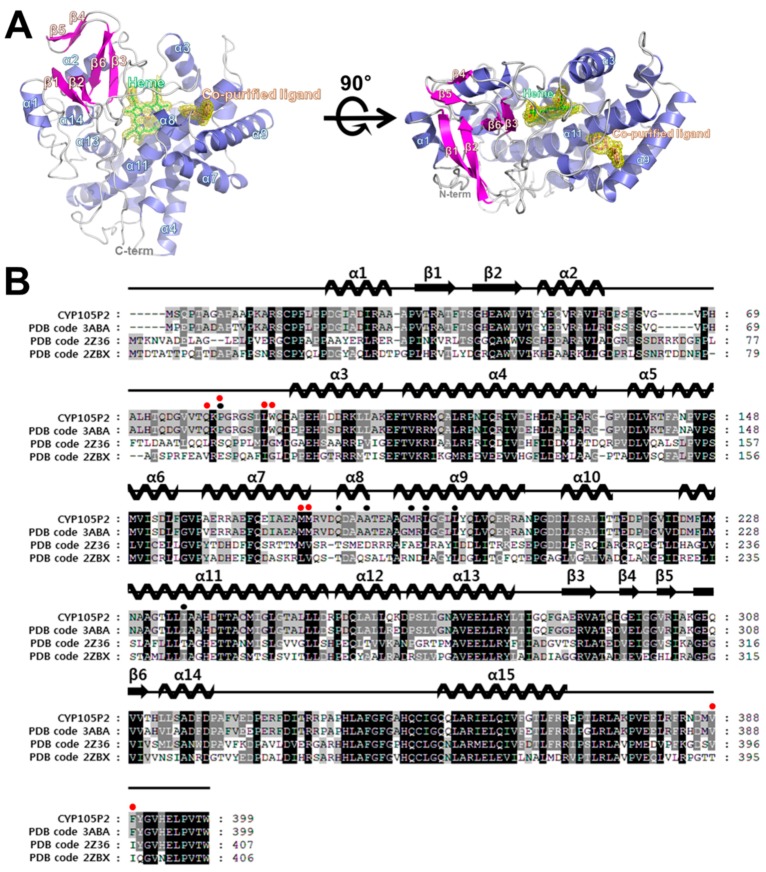
Crystal structure of CYP105P2. (**A**) Overall structure of CYP105P2 is shown as ribbon diagram with α-helices colored slate blue and β-strands colored magenta. Bound heme molecule and co-purified ligand (orange) are shown as stick models with a (2*F*_o_ − *F*_c_) electron density map (contoured at 1*σ*). N- and C-termini are labeled; (**B**) Multiple sequence alignment of CYP105P2, CYP105P1 (PDB code: 3ABA; UniProtKB code: Q93H81), CYP105 (structurally similar to CYP55A1) (PDB code: 2Z36; UniProtKB code: Q2L6S8), and CYP105A1 (PDB code: 2ZBX; UniProtKB code: P18326). Secondary structural elements in the crystal structure of CYP105P2 are represented above the multiple-sequence alignment. The highly conserved residues are shaded in black, and residues not fully conserved are marked by gray boxes. Residues participating in co-purified ligand interactions of CYP105P2 and filipin I-bound CYP105P1 structure (PDB code 3ABA) are indicated above the alignment residues with black circles and red circles, respectively. Multiple-sequence alignment was performed using ClustalX (version 1.81) [30] and edited with GeneDoc (Ver 2.5.000) [31].

The structure of CYP105P2 from *S. peucetius* was determined at a resolution of 2.1 Å. It contains 15 α-helices and six β-strands (Figure 2A). The putative active site of CYP105P2 is composed of a heme group and hydrophobic substrate-binding site. The heme group is tightly bound in the middle of the CYP105P2 protein, therefore its atoms have the lowest *B* factors (33.2 Å^2^) in the structure. The amino acids that contact the heme group are highly conserved among the known structures of different CYPs. In detail, the heme O1A and O1D interact with the side chain of Arg290. The heme O2A is hydrogen bonded to the ND1 of His346 and the heme O1A is hydrogen bonded to the NE2 of His95. The heme O1A forms an additional hydrogen bond with the NH1 of Arg99. The ring structure of heme forms tight hydrophobic interactions with the Leu88, Ala237, Ala238, Phe341, and Ile349 side-chains.

Although no exogenous ligands were added to the CYP105P2 protein during purification or crystallization, initial experimental maps indicated the presence of an unknown ligand near the active sites of both molecules in the asymmetric unit. A careful examination of the (*F*_o_ − *F*_c_) difference density map following refinement indicated that there was a distinct electron density with a bent V-shape. The conformation of this density suggests that it could be interpreted as a biphenyl derivative, which was then fitted into this electron density map. The bound ligand forms strong hydrophobic interactions with Pro82, Ala181, Met187, Leu189, Leu193, and Ile236 side-chains. In addition, the hydroxyl group of the biphenyl molecule forms hydrogen-bonding interactions with the NE2 of Gln177. Notably, all of these interacting residues are strictly conserved between CYP105p1 and CYP105P2. However, the ligand-binding residues (Gln80, Pro82, Leu88, Trp89, Met172, Met173, Val388, and Phe389) in the filipin I-bound CYP105P1 structure (PDB code 3ABA) significantly differed from the co-purified ligand-interacting residues of CYP105P2 (Figure 2B).

### 2.4. Comparison of Ligand-Bound CYP105P2 Structure with Other CYP Structures

**Figure 3:**
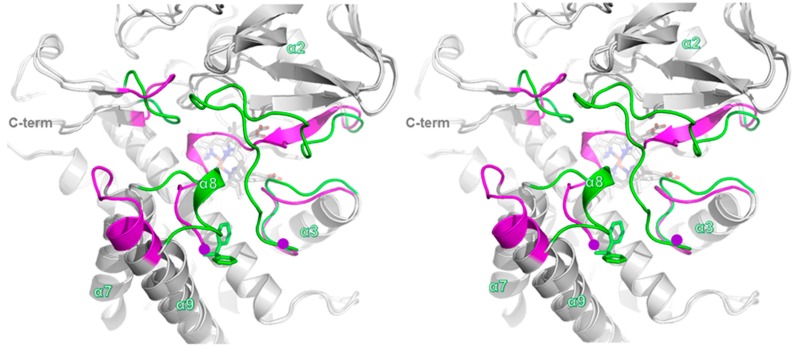
Stereo view of superimposed structures of CYP105P2 complexed with co-purified ligand (biphenyl compound) and ligand-free CYP105P1 (PDB code 3E5J). Conformational differences in the α8 region loop (residues 175–183), *C*-terminal loop region (385–390), and α2–α3 loop region (61–92) are shown in CYP105P2. Loop structure of CYP105P2 in co-purified ligand-bound form is shown in green, and loop structure of *apo*-form CYP105P1 is shown in magenta. Co-purified ligand in CYP105P2 molecule is represented by green sticks.

**Figure 4:**
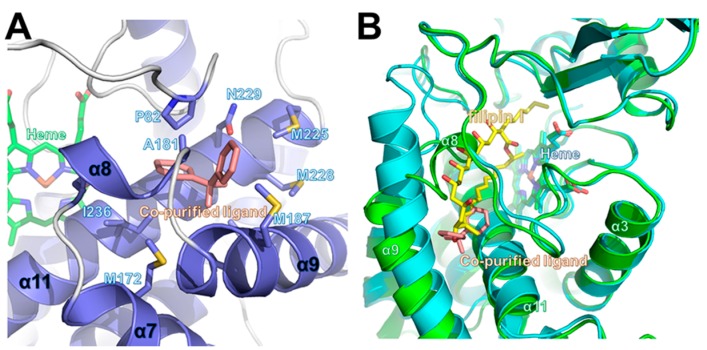
Ligand-binding site of CYP105P2. (**A**) Interactions between co-purified ligand and CYP105P2; (**B**) Structural superimposition of CYP105P2 structure bound to co-purified ligand (salmon), and CYP105P1 (PDB code: 3ABA; cyan) bound to filipin I (yellow). Partially overlapping ligand-binding site is shown.

Despite the high sequence similarity (over 90% sequence identity), a comparison of the structures of CYP105P2 and *apo*-CYP105P1 (PDB code 3E5J) highlights significant differences between the outer loop regions of their substrate-binding cavities (Figure 3). In detail, these divergent regions include the α8 region loop (residues 175–183), *C*-terminal loop region (385–390), and α2–α3 loop region (61–92) in CYP105P2. In the absence of a substrate, the α8 region loop of CYP105P1 is located in the substrate-binding pocket, covering the cavity. Moreover, the α7–α9 loop region residues (79–82) are partially disordered in the *apo*-CYP105P1 structure; this is accounted for by the fact that this region of CYP105P1 is mobile in the absence of a ligand (Figure 4A). The CYP105P1 substrate is a large macrolide molecule known as filipin I. Notably, the filipin I-bound CYP105P1 structure differs significantly from CYP105P2 in these loop regions. After substrate binding, the filipin I-bound CYP105P1 structure (PDB code 3ABA) adopts a more extended α9 helix and shorter α7–α9 loop compared with those of CYP105P2. These structural differences provide enough pocket space to accommodate filipin I binding (Figure 4B). These results indicate that the region is mobile and can be altered by ligand interactions.

In previous studies of the enzymatic kinetics of CYP105P2 performed using aromatic compounds, we established the preference of CYP105P2 for the specific hydroxylation of flavones [23]. Furthermore, a homology model of flavone-bound CYP105P2 led to the proposal that the flavone-interacting residues would be Leu88, Trp89, Pro82, Ala184, Ile236, Ala237, and Thr241 [25]. A comparison of the pseudo-ligand (biphenyl)-bound CYP105P2 crystal structure and the homology model of flavone-bound CYP105P2 highlighted several structural differences in the ligand-binding pocket. These results support the conclusion that CYP105P2 has distinctive, ligand-specific structural features within the ligand-binding sites. Although the global folds and heme-binding motif structures of CYPs are similar to each other, the differences between the sequences and structures of their ligand-binding sites may contribute to their ability to recruit their distinct substrates.

A structural similarity search was carried out with the DALI program [32] using the coordinates of the CYP105P2 structure. The top nine DALI hits were selected (Table 1) and compared using structural superposition. In this structural comparison, we observed that the bending and movement of the α8 and α9-helix is essential for large-ligand binding. It was apparent from analysis of the MycG-mycinamicin [33] and CYP107W1-Oligomycin A complex structures that the helix bending facilitates bulky substrate entry by enlarging the internal cavity of the substrate pocket. This result also supports the idea that the region around the α8-helix is the functionally evident portal of substrate entry in other CYPs.

## 3. Experimental Section

### 3.1. Purification of Streptomyces peucetius CYP105P2

Cloning and heterologous expression of *Streptomyces peucetius* CYP105P2 was carried out according to our previously published methods [23,29]. The protein was purified by a metal affinity method with Ni^2+^–NTA resin (Qiagen, Hilden, Germany). The polyhistidine-tagged CYP105P2 bound to the resin was washed with ten bed column volumes of washing buffer (50 mM sodium phosphate, 300 mM sodium chloride, 20 mM imidazole, pH 7.4). The protein was eluted with elution buffer (50 mM sodium phosphate, 300 mM sodium chloride, 300 mM imidazole, pH 7.4). The eluted purified protein was concentrated using Amicon Ultra centrifugal filters (Ultracel-3K; Millipore, Darmstadt, Germany). The polyhistidine tag was cleaved by thrombin during overnight incubation at 4 °C. The sample was then loaded onto a Superdex-200 column (GE Healthcare, Piscataway, NJ, USA), which had been equilibrated with 150 mM sodium chloride, 20 mM Tris–HCl, pH 7.4. The fractions containing red-colored CYP150P2 were collected, and then concentrated to 6.63 mg/mL by centrifugal filtration.

### 3.2. Crystallization and Data Collection

Various commercial crystallization solution kits were used for initial crystal screening (MCSG I–IV (Microlytic, Burlington, VT, USA); Wizard Classic I–IV (Emerald Bio, Seattle, DC, USA). Crystallization of CYP105P2 was performed using the sitting-drop vapor-diffusion method at 293 K in 96-well crystallization plates (Emerald Bio, Bainbridge Island, WA, USA). A 6.63 mg/mL solution of CYP105P2 was mixed with equal volume of reservoir solution. Crystals of CYP105P2 with a bipyramidal morphology appeared within 3 days when the reservoir solution contained 0.1 M bis-Tris propane, pH 7.0, and 0.2 M dl-malic acid, pH 7.0 (MCSG-4 #B10). A single crystal was harvested directly from the 96-well crystallization plate and cryo-protected using Paratone-N oil (Hampton Research, Aliso Viejo, CA, USA); it was stored in liquid nitrogen. A diffraction data set was collected at the BL-7A beam line of the Pohang Accelerator Laboratory (PAL; Pohang, Korea) using an X-ray wavelength of 0.97934 Å. The data set contained 360 images, which were then processed, integrated, and scaled using the HKL-2000 software suite [37]. The data collection statistics are summarized in Table 2.

### 3.3. Structure Determination and Refinement of CYP105P2

The data set contained 360 images at a 2.1 Å resolution. The CYP105P2 crystal belongs to the *C*2, space group, with unit cell parameters *a* = 122.633 Å, *b* = 122.864 Å, *c* = 193.090 Å, *α* = 90.00°, *β* = 90.02°, and *γ* = 90.00°. The CYP105P2 structure was determined by molecular replacement using the MOLREP program [38], in the CCP4 program suite [39]. The CYP105P1 crystal structure from *S. avermitilis* (PDB code 3ABA) [21] was used as the search model. The model was built manually in COOT [40], and the model was refined using REFMAC5 [41]. The *R*_cryst_ and *R*_free_ values of the final refined structure were 0.202 and 0.243, respectively. Further details pertaining to the structure determination and refinement are summarized in Table 2. All structural diagrams of CYP105P2 were prepared using PyMOL [42]. The final atomic coordinates of CYP105P2 were deposited in the Protein Data Bank under the accession code 5IT1.

## 4. Conclusions

Structural analysis of CYP105P2 and comparison of its structure with those of other ligand-complexed CYPs enabled conclusive assignment of ligand-binding sites and structural flexibilities to CYP105P2 on binding of different ligands. Several hydrophobic residues (Pro82, Leu189, Leu193, and Ile236) in the hydrophobic ligand-binding cleft seem to be used invariantly to interact with various ligands. Several residues located in the lid loops of the substrate channel are flexible, changing orientation on insertion of different ligands. This suggests the presence of residues specifically clustered in the lid loops that can discriminate between the insertion of different ligands into the hydrophobic catalytic cleft. The architecture of the lid loops appears to be structurally optimized to attract different ligands into the catalytic cleft. The structural plasticity seen in the lid loops can explain the remarkably broad substrate specificity of CYP105P2. Further understanding of the substrate specificity and molecular mechanisms in the various reactions performed by CYPs would facilitate their potential use in site-specific biocatalysis. The synthesis of site-directed hydroxylated compounds is important for drug design, because such modifications can increase drug solubility and alter its toxicological profile.

## Figures and Tables

**Table 1:** Selected structural homologues of CYP105P2 from a DALI search (DALI Lite version 3 server).

Protein	PDB Code	DALI Score	Sequence % ID (Aligned Residue Number/Total Residue Number)	Reference
CYP105P1	3ABA	60.0	92% (385/397)	[21]
MycG	3ZSN	50.1	37% (380/393)	[33]
MoxA	2Z36	49.9	37% (380/393)	[26]
CYP105AS1	4OQS	49.7	44% (368/384)	[34]
OleP	4XE3	48.7	37% (378/394)	[35]
CYP107W1	4WQ0	48.4	38% (375/397)	It has not yet been published
CYP105N1	3TYW	47.3	41% (379/399)	It has not yet been published
CYP105A1	3CV8	46.8	42% (379/402)	[36]
CYP105D6	3ABB	46.7	39% (371/383)	[21]

**Table 2:** Data collection and refinement statistics.

Data Set	CYP105P2 + Biphenyl Molecule
X-ray source	PAL 7A beam line
Space group	*C*2
Wavelength (Å)	0.97934
Resolution (Å)	50.00–2.10 (2.14–2.10)
Total reflections	1146063
Unique reflections	165620 (8315)
Average *I*/*σ* (*I*)	39.3 (4.6)
*R*_merge_^a^	0.104 (0.823)
Redundancy	6.9 (7.1)
Completeness (%) ^b^	99.5 (100.0)
**Refinement**	
Resolution range (Å)	50.0–2.10 (2.16–2.10)
No. of reflections in working set	157,003 (11,120)
No. of reflections in test set	8315 (564)
No. of amino acid residues	1580
No. of water molecules	657
*R*_cryst_^b^	0.200 (0.317)
*R*_free_^c^	0.243 (0.352)
RMS bond length (Å)	0.019
RMS bond angle (°)	1.927
Average *B* value (Å^2^) (protein)	48.510
Average *B* value (Å^2^) (solvent)	51.306

^a^ *R*_merge_ = ∑｜<*I*> − *I*｜/∑<*I*>; ^b^ *R*_cryst_ = ∑｜|*F*_o_| − |*F*_c_|｜/∑|*F*_o_|; ^c^ *R*_free_ calculated with 5% of all reflections excluded from refinement stages using high-resolution data; Values in parentheses refer to highest resolution shells.

## Abbreviations

The following abbreviations are used in this manuscript:

CYP	Cytochrome P450
ALA	5-α-aminolevulinic acid
MCS	multiple-cloning site
